# Impact of Acetic Acid on the Survival of *L. plantarum* upon Microencapsulation by Coaxial Electrospraying

**DOI:** 10.1155/2017/4698079

**Published:** 2017-07-05

**Authors:** Laura G. Gómez-Mascaraque, Jesús Ambrosio-Martín, Rocío Perez-Masiá, Amparo Lopez-Rubio

**Affiliations:** Food Quality and Preservation Department, IATA-CSIC, Avda. Agustin Escardino 7, Paterna, 46980 Valencia, Spain

## Abstract

In this work, coaxial electrospraying was used for the first time to microencapsulate probiotic bacteria, specifically *Lactobacillus plantarum*, within edible protein particles with the aim of improving their resistance to in vitro digestion. The developed structures, based on an inner core of whey protein concentrate and an outer layer of gelatin, were obtained in the presence of acetic acid in the outer solution as a requirement for the electrospraying of gelatin. Despite the limited contact of the inner suspension and outer solution during electrospraying, the combination of the high voltage used during electrospraying with the presence of acetic acid was found to have a severe impact on the lactobacilli, not only decreasing initial viability but also negatively affecting the survival of the bacteria during storage and their resistance to different stress conditions, including simulated in vitro digestion.

## 1. Introduction

Probiotics can be incorporated into food products as bioactive ingredients for the development of functional foods. For this purpose, the microorganisms must be alive and metabolically active, and their concentration at the time of consumption should be high enough to exert their claimed health benefits [1]. Therefore, stabilization of bacteria is of outmost importance when supplementing food products with sensitive probiotic cultures whose survival can be compromised during their shelf life or digestion, and microencapsulation technologies are regarded as an effective approach to achieve it [2–4].

Electrospraying is a versatile electrohydrodynamic processing technique which can be used to generate ultrafine polymeric particles in a one-step process under mild conditions [5] by applying a high-voltage electric field to a polymer-containing fluid, causing its spraying towards a grounded collector where dry material is deposited [6–8]. This technology can be used for the microencapsulation of bioactive agents as an alternative to conventional techniques [9].

In a previous work, we showed the potential of this technique for the microencapsulation of *L. plantarum* within whey protein concentrate (WPC) capsules [10]. Although the obtained capsules proved to better protect the bacterial viability during storage under stress conditions than a traditional preservation technique such as freeze-drying, the protection exerted during simulated digestion was found to be similar using both techniques. This was attributed to the water-dispersible nature of the protein matrix used for encapsulation, which led to the disruption of the capsules in aqueous environments.

In an attempt to broaden the application range of the previously developed carriers to aqueous food products and enhance their protective effect during digestion, gelatin was selected in this work as a hydrogel-forming protein to coat the probiotic-loaded WPC particles. Gelatin can be electrosprayed from aqueous solutions while avoiding the complete disruption of the obtained capsules in aqueous environments below its gel-sol transition temperature but requires dissolution in diluted acid for electrospraying [11].

Coaxial electrospraying is a specific electrospraying configuration which allows the simultaneous spraying of two different liquids from two concentric capillaries, so that a core liquid flows from the central capillary and the other fluid is pumped through the external, annular space between both capillaries [12]. This approach has already been employed for the encapsulation of bioactive agents for pharmaceutical applications [13–15] and has very recently been proposed for the encapsulation of lycopene for food applications [16].

In this work, a coaxial electrospraying configuration was used to obtain gelatin-coated WPC capsules containing *L. plantarum*. The protective ability of the developed structures during storage, under stress conditions, and during in vitro digestion was evaluated, and the impact of the acetic acid on the survival of microencapsulated *L. plantarum* is reported.

## 2. Materials and Methods

### 2.1. Materials

*Lactobacillus plantarum* strain CECT 748 T was obtained from the Spanish Cell Culture Collection (CECT) and routinely grown in Man, Rogosa, and Sharpe (MRS) broth (Scharlau, Barcelona, Spain). Serial dilutions were made in 1% meat peptone solution, and plate counting was performed on MRS agar, both provided by Conda Pronadisa (Spain). Whey protein concentrate (WPC), under the commercial name of Lacprodan® DI-8090, was kindly donated by ARLA Food Ingredients (Viby, Denmark). Type A gelatin from porcine skin (175 g Bloom), pepsin from porcine gastric mucosa, pancreatin from porcine pancreas, bile extract porcine, and phosphate-buffered saline (PBS) were purchased from Sigma-Aldrich (Spain). All inorganic salts used for the in vitro digestion tests were used as received.

### 2.2. Preparation of Feed Solutions/Dispersions

Gelatin solutions (5% *w*/*v*) in acetic acid (10% *v*/*v*) were prepared as previously described in Gómez-Mascaraque et al. [11]. WPC dispersions (30% *w*/*v*) in distilled water were prepared as described in Gómez-Mascaraque et al. [10]. The probiotic bacteria suspensions were prepared by incorporation of a fresh culture of *L. plantarum* within the WPC dispersions following the protocol described in Gómez-Mascaraque et al. [10].

### 2.3. Preparation of Probiotic-Containing Capsules through Electrospraying

The suspensions were processed by coaxial electrospraying through two concentric stainless-steel needles (0.6 and 1.4 mm of inner diameters, resp.) using a Fluidnatek® LE-10 electrospinning/electrospraying apparatus, equipped with a variable high-voltage 0–30 kV power supply. Probiotic-containing WPC suspensions were pumped from a sterile 5 mL plastic syringe at 0.05 mL/h through the inner needle, and gelatin solutions were pumped from an identical syringe at 0.15 mL/h through the outer needle, using two digitally controlled syringe pumps (KD Scientific Inc., Holliston, USA). The suspensions were processed at an applied voltage of 17 kV, and dry materials were collected on a stainless-steel plate connected to the ground electrode of the power supply and placed at a distance of 10 cm with respect to the tip of the needle. For comparison purposes, a uniaxially electrosprayed material was also obtained under the same conditions by pumping only the probiotic-containing WPC suspensions through the inner needle at 0.05 mL/h and referred to as “single-fluid” electrosprayed samples.

### 2.4. Morphological Characterization of the Microcapsules

Scanning electron microscopy (SEM) was conducted on a Hitachi microscope (Hitachi S-4800) following the method described in Gómez-sMascaraque et al. [10].

### 2.5. Viability of Free and Encapsulated *L. plantarum*

The viability of *L. plantarum* was evaluated by plate counting on MRS agar as described in Gómez-Mascaraque et al. [10]. The number of colony-forming units (CFU) per unit mass of WPC was determined after 24–48 h incubation at 37°C for the probiotic-containing WPC suspensions before processing and in the dry electrosprayed products, by resuspension of the latter in peptone solution and subsequent dissolution of the gelatin coating by mild heating at 37°C. All tests were made in triplicate.

### 2.6. Survival of Encapsulated *L. plantarum* during Storage and under Stress Conditions

The coaxially electrosprayed capsules containing *L. plantarum* were stored in a freezer for one month, after which the bacterial viability was tested. For comparison purposes, *L. plantarum*-containing WPC capsules were also prepared by uniaxial electrospraying and were subjected to the same storage conditions.

The WPC-gelatin capsules were also subjected to different stress conditions, and the resistance of the probiotic strain was tested. For this purpose, the survival of *L. plantarum* was evaluated after heating the materials at 120°C. In addition, the microparticles were stored at high relative humidity (75%) and bacterial viability drop was evaluated with storage time. For comparison purposes, *L. plantarum*-containing WPC capsules were also prepared by uniaxial electrospraying under the optimal conditions determined in Gómez-Mascaraque et al. [10] and were subjected to the same stress conditions.

### 2.7. Survival of Encapsulated *L. plantarum* during In Vitro Digestion

Suspensions (30 mg/mL) of the electrosprayed capsules in distilled water were subjected to in vitro gastrointestinal digestion according to the protocol described in Gómez-Mascaraque et al. [10], which is based on a standardized static in vitro digestion protocol [17]. Aliquots were collected after the gastric and the duodenal phases, and the viability of *L. plantarum* in the digester was assessed by plate counting.

### 2.8. Assessment of Residual Acid within the Electrosprayed Capsules

The core-shell WPC-gelatin capsules were suspended in distilled water at 2 mg/mL and disrupted by mild heating at 37°C during 2 h under frequent and vigorous agitation. A mixture of raw gelatin and WPC containing the same protein mass ratio as the electrosprayed material was equally dispersed in water at the same concentration. The pH of both suspensions was measured using a pH-meter PB-11 (Sartorius, Spain), and both results were compared.

### 2.9. Statistical Analysis

Statistical analysis of experimental data was performed using IBM SPSS Statistics software (v.23) (IBM Corp., USA). Significant differences between homogeneous sample groups were obtained through two-sided *t*-tests at the 95% significance level.

## 3. Results and Discussion

### 3.1. Morphology of the Electrosprayed Microcapsules

**Figure 1:**
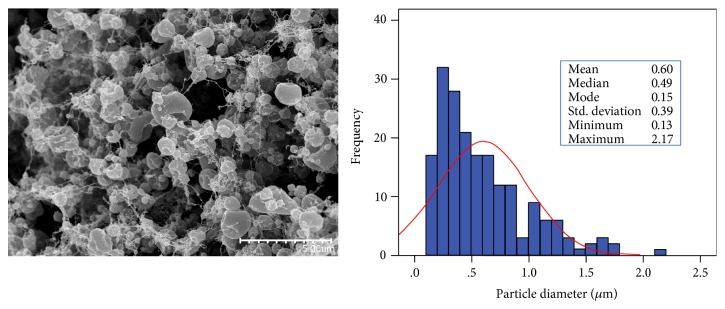
SEM micrograph of coaxially electrosprayed *L. plantarum*-loaded encapsulation structures based on gelatin and WPC and their particle size distribution. Scale bar corresponds to 5 *μ*m.

With the aim of making WPC capsules more resistant to disruption in aqueous environments, bacteria-containing WPC cores were coated with an outer gelatin layer by coaxial electrospraying. For this purpose, the WPC suspension containing *L. plantarum* and the gelatin solution were pumped through the inner and outer needles, respectively, and subjected to electrohydrodynamic processing according to the method described in Sections 2.2 and 2.3.  Figure 1 shows a SEM micrograph of the obtained material, together with its particle size distribution.

The material exhibited a particulate morphology, with pseudo-spherical shapes, similar to that obtained in a previous work for uniaxially electrosprayed gelatin capsules [11]. However, the particle diameters were bigger, presumably due to the incorporation of WPC (plus bacteria) in the core of the capsules. These bigger sizes can also be favourable for the incorporation of the cells within the capsules.

**Figure 2:**
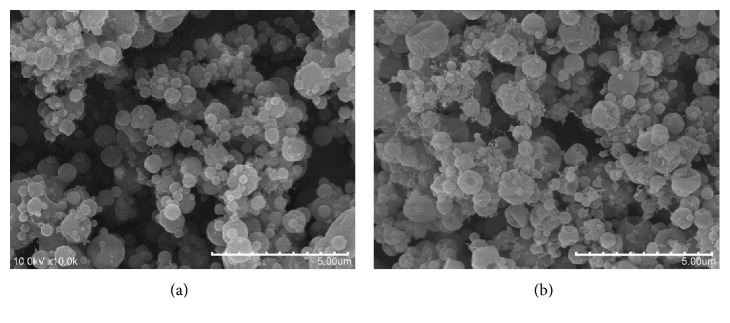
Morphology of gelatin-WPC capsules obtained using 30% (a) and 40% (b) acetic acid (*v*/*v*) to electrospray the gelatin solutions. Scale bar corresponds to 5 *μ*m.

Also noticeable is the presence of ultrafine fibers within the material. In a preliminary optimization of the electrospraying conditions, it was observed that by increasing the acetic acid content in the gelatin solution to 30% (*v*/*v*), mostly neat particles with almost no traces of fibers were obtained (Figure 2). The solvent used has a great impact on the morphology of the materials obtained through electrospraying, not only because its volatility determines the drying rate during the process [8] but also because its characteristics affect solution properties such as the conductivity, the surface tension, or the viscosity, which are key parameters influencing the performance of electrospraying processes [18]. For the WPC-gelatin coaxial system explored in this work, higher acetic acid concentrations in the gelatin solution yielded less fibrillar microstructures, as observed in Figure 2. However, although the contact of the outer and inner polymer dispersions in the tip of the needle during coaxial electrospraying is short, this high concentration of acetic acid in the gelatin solution led to a complete viability loss of the encapsulated cells. Therefore, an acetic acid concentration of 10% (*v*/*v*) was finally selected, as it yielded acceptable bacterial viabilities in the just-produced capsules (see below).

### 3.2. Viability of Microencapsulated *L. plantarum*

Although high voltages may have a biocidal effect under certain conditions, due to atmospheric corona discharges [19], the conditions of the electrospraying process did not significantly affect the viability of some commercial probiotics, such as *Bifidobacterium animalis* subsp. *Lactis* Bb12 [20]. However, certain viability loss (lower than 1 log_10_ CFU/g) did occur upon microencapsulation of *L. plantarum* in our previous work [10].

The viability of *L. plantarum* within the electrosprayed powders obtained in this work, expressed per unit mass of WPC, is shown in Table 1. The loss in CFU during electrospraying was somewhat higher than that obtained in the previous work in the absence of gelatin [10]. Since the processing conditions were similar, these greater losses could be attributed to the presence of acetic acid in the gelatin solution. In order to corroborate this hypothesis, lactobacillus-loaded WPC capsules were produced using exactly the same conditions as for the coaxial electrospraying, including the configuration of the coaxial circuit, but pumping only the inner solution. The bacterial viabilities of the materials obtained by this procedure, referred as “single-fluid” processing in Table 1, were higher than those containing gelatin and consistent with the previous work [10].

Preliminary tests showed that increasing the concentration of acetic acid had a negative impact on the viability of *L. plantarum* in the electrosprayed product (see previous section), revealing that despite the short contact of the gelatin solution with the bacteria-containing WPC suspension at the tip of the needle, the acetic acid must have diffused through the latter, having an impact on the probiotic viability. It has already been reported that the viability of microencapsulated *Lactobacillus* species was greatly reduced when the electrosprayed capsules were coated with acidified zein [21]. In that study, however, the core suspension containing the bacteria was directly electrosprayed into the acid shell solution, taking several hours to dry the particles. By the coaxial electrospraying approach, the time of contact between the core and shell fluids before drying is minimized, so a reduced impact was expected. Indeed, the viability of *L. plantarum* within the coaxially electrosprayed capsules was still high, surpassing 8 log_10_ CFU/g even for the longest processing times.

### 3.3. Survival of Microencapsulated *L. plantarum* during Storage and under Stress Conditions

**Figure 3:**
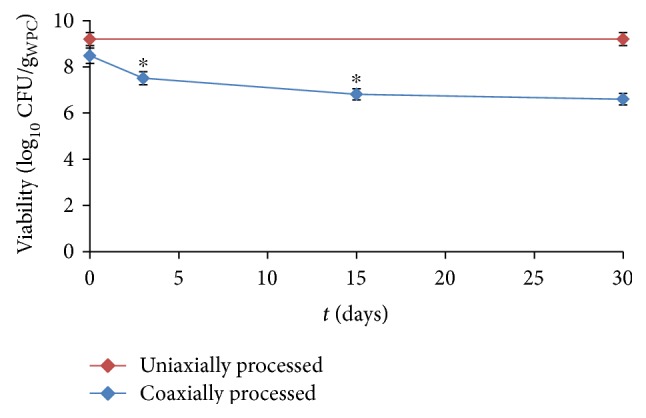
Viability of *L. plantarum* within the electrosprayed microcapsules during storage in the freezer. Asterisk (^∗^) depicts significant differences for one sample with respect to the previous time point at *p* < 0.05.

The viability of *L. plantarum* within the coaxially electrosprayed microencapsulation structures was evaluated during storage in the freezer, and the results are shown in Figure 3, in comparison with the viability of a uniaxially processed sample. While the latter remained stable during one month of storage, a reduction in the bacterial viability was observed for the gelatin-coated structures, especially during the first days. In the light of these results, it was hypothesized that, apart from causing an initial loss in CFU, acetic acid could have damaged viable bacterial cells in such a way that their death was accelerated during storage. This damage might have been magnified in combination with the osmotic shock suffered by the microorganisms during the electrospraying process. Alternatively, or simultaneously, residual traces of acetic acid could remain trapped within the gelatin-containing capsules after processing, despite the fast drying of the structures during electrospraying. The inhibition of the growth of some lactobacillus species in the presence of acetate in aerobic conditions has long been reported [22].

Culturability of the probiotic strain was also evaluated after subjecting the capsules to different stress conditions, that is, storage at high temperature (120°C) and high relative humidity (75% RH), in order to assess the protective ability of the materials. The results were compared to those obtained when *L. plantarum* was encapsulated through the optimized procedures described in Gómez-Mascaraque et al. [10]. Whereas the uniaxially electrosprayed samples still showed a viability of 5.4 ± 0.4 and 4.9 ± 0.1 log_10_ CFU/g, respectively, after 15 min at 120°C, no viable bacteria were detected after the same time period for the gelatin-coated capsules, meaning that the probiotic strain could not be protected against such a high temperature when encapsulated within these core-shell WPC-gelatin structures. Similar results were obtained upon storage at high relative humidities. After only three days of storage at 75% RH, no viable cells were detected by plate counting in the coaxially electrosprayed samples, while viable bacteria were still detected in the uniaxially electrosprayed materials after 10 days [10]. These results emphasise the detrimental impact of acetic acid on the probiotic strain.

### 3.4. Presence of Residual Acetic Acid within the Electrosprayed Microcapsules

In order to corroborate the presence of remaining acetic acid within the microstructures, these were disrupted in distilled water at 37°C and the pH of the dispersion was compared to that of an equivalent mixture of raw gelatin and WPC. Indeed, while the pH of the first dispersion was ~4, the mixture of raw proteins had a pH greater than 5, thus evidencing the presence of residual acetic acid within the electrosprayed material. This acidic environment might have contributed to the accelerated decay in bacterial viability during storage and under stress conditions.

### 3.5. Survival of Microencapsulated *L. plantarum* during In Vitro Digestion

The viability of coaxially microencapsulated *L. plantarum* was also evaluated after in vitro digestion. Despite the initial hypothesis that a hydrogel coating would increase the protection of the probiotic strain in aqueous environments, including simulated digestion conditions, through limited capsule disruption [23], no culturable bacteria were detected after the gastric phase. Although it has been previously observed that the main loss in CFU for this strain takes place after the gastric phase, only viability drops lower than 2 log_10_ CFU/g were found when it was encapsulated within WPC-based particles in the absence of gelatin [10]. Thus, the proposed coaxial approach not only did not enhance the protection of the probiotic microorganisms during digestion but clearly deteriorated it.

Given that uniaxially encapsulated bacteria better survived in a simulated gastric environment (i.e., pH = 3) than their coaxially encapsulated counterparts, the hypothesis of an irreversible damage during the electrospraying process seems more plausible, as the potential effect of the residual acetic acid within the capsules would be overshadowed by the already low pH of the suspension medium in this assay. Although the impact of acetic acid could be relevant during dry or humid storage, a great difference in the survival of *L. plantarum* was also observed between both types of capsules when suspended in a very acidic medium, supporting the idea that bacteria might have been irreversibly damaged before the assay. These bacteria would then have been weakened during the electrospraying process itself, by the combined effect of the osmotic shock caused by the rapid drying (which also occurs during uniaxial electrospraying) together with the presence of acetic acid.

The results obtained by Laelorspoen et al. [21] for other species of lactobacilli (*L. acidophilus*) also support this hypothesis. In their work, the cells were subjected to acidic pH only after the electrospraying process (which was also carried out at much lower voltages), as the electrosprayed alginate particles were ejected into an acidic zein bath once formed. Although the initial cell counts were greatly affected by the concentration of acid in the zein solution, the core-shell capsules demonstrated enhanced protection of the lactobacilli in simulated gastric fluid, so no apparent cell damage was caused to the culturable cells which survived the encapsulation process. In contrast, *L. plantarum* in our work was in contact with the acid during the electrospraying process itself, that is, during the osmotic shock they suffered, apparently suffering severe cell damage.

## 4. Conclusions

*L. plantarum*-containing gelatin-WPC microcapsules were produced by coaxial electrospraying for the first time. Despite the limited contact of the acidic gelatin solution with the bacterial suspension at the tip of the concentric needles, the loss in CFU of *L. plantarum* increased during electrospraying as compared to the uniaxial process in the absence of gelatin. Moreover, a rapid reduction in bacterial counts was observed soon after production during storage in the freezer, and the gelatin-coated structures were unable to protect *L. plantarum* from thermal stress, high-humidity environments, or during digestion, while uniaxially electrosprayed WPC-capsules had shown enhanced survival rates. These results led to the conclusion that acetic acid had a worse impact on cell viability than expected. Not only did it cause an initial loss in CFU but it also seemed to irreversibly damage viable bacteria, accelerating its decay during storage and making them more vulnerable to stress conditions. This damage was believed to be a consequence of the combined effect of the low pH and the osmotic shock to which the cells are subjected during electrospraying. Also, residual acetic acid was found to remain trapped within the electrosprayed microcapsules, possibly impacting the cell survival too. Further research is needed to gain insight on the precise mechanisms which cause cell damage during electrospraying in the presence of acids.

## Figures and Tables

**Table 1:** Viability of *L. plantarum* in the electrosprayed materials and loss in CFU experienced during processing. Different letters (a-b) within the same column indicate significant differences at *p* < 0.05 among the samples.

	Viability (log_10_ CFU/g)	Loss in CFU (log_10_ CFU/g)
After coaxial processing	8.5 ± 0.1^a^	1.4 ± 0.2^a^
After “single-fluid” processing	9.4 ± 0.1^b^	0.5 ± 0.2^b^

## Abbreviations

CECT: Spanish Cell Culture Collection
MRS: Man, Rogosa, and Sharpe culture medium
RH: Relative humidity
SEM: Scanning electron microscopy
WPC: Whey protein concentrate.
